# High Neutrophil Percentage-To-Albumin Ratio Can Predict Occurrence of Stroke-Associated Infection

**DOI:** 10.3389/fneur.2021.705790

**Published:** 2021-09-08

**Authors:** Haipeng Zhang, Ti Wu, Xiaolin Tian, Panpan Lyu, Jianfei Wang, Yang Cao

**Affiliations:** ^1^Department of Clinical Laboratory, The Second Hospital of Tianjin Medical University, Tianjin, China; ^2^Department of Neurology, Tianjin Medical University General Hospital, Tianjin, China; ^3^Department of Neurology, The Second Hospital of Tianjin Medical University, Tianjin, China; ^4^Department of Medical Laboratory, Clinical Medical College of Tianjin Medical University, Tianjin, China

**Keywords:** stroke, infection, neutrophil, albumin, inflammation

## Abstract

**Purpose:** Stroke-associated infection (SAI) is associated with adverse outcomes in patients with acute ischemic stroke (AIS). In this study, we aimed to evaluate the association between neutrophil percentage-to-albumin ratio (NPAR) and SAI occurrence in patients with AIS.

**Methods:** We retrospectively analyzed all AIS patients who were admitted to the Neurology ward of The Second Hospital of Tianjin Medical University from November 2018 to October 2020. The relationship between NPAR and SAI was analyzed by multivariable analysis. The receiver operating characteristic (ROC) curve was used to compare the predicted value of albumin, neutrophil percentage, neutrophil-to-lymphocyte ratio (NLR), and NPAR.

**Results:** We included 379 AIS patients out of which 51 (13.5%) developed SAI. The NPAR was independently associated with increased risk of SAI adjusting for confounders [adjusted odds ratio (aOR) = 10.52; 95% confidence interval (CI), 3.33–33.28; *P* <0.001]. The optimal cutoff value of NPAR for predicting SAI incidence was 1.64, with sensitivity and specificity of 90.2 and 55.8%, respectively. The area under the curve (AUC) value of NPAR [0.771 (0.725–0.812)] was higher than that of albumin [0.640 (0.590–0.689)], neutrophil percentage [0.747 (0.700–0.790)], and NLR [0.736 (0.689–0.780)], though the statistical significance appeared only between NPAR and albumin.

**Conclusions:** We demonstrated that a higher NPAR could predict the occurrence of SAI. Thus, NPAR might be a more effective biomarker to predict SAI compared with albumin, neutrophil percentage, and NLR.

## Introduction

Stroke-associated infection (SAI) is one of the most common complications in patients with acute ischemic stroke (AIS) (1, 2). It has been reported that pneumonia and urinary tract infections are the most prevalent SAIs (3–5). SAI considerably increases disability and length of hospital stay for patients with AIS. Furthermore, it is one of the leading causes of death (6, 7). Early diagnosis and treatment are the best-known ways to reduce the SAI risk; hence, a simple and effective biomarker is needed to predict SAI.

Neutrophil percentage-to-albumin ratio (NPAR) is a novel indicator of systemic inflammation and infection. Several studies have shown that NPAR could be used as a prognostic indicator for patients with cardiogenic shock, myocardial infarction, acute kidney injury, and cancer (8–11). Additionally, it is well-known that high neutrophil percentage predicts bloodstream infection, while low albumin levels increase the susceptibility to infection complications. However, the relationship between NPAR and SAI is rarely reported to date. We aimed to explore the role of NPAR in predicting SAI in patients with AIS.

## Materials and Methods

### Study Population

This was a retrospective study and approved by the Ethics Committee of The Second Hospital of Tianjin Medical University. All patients with AIS who were admitted to the Neurology ward of The Second Hospital of Tianjin Medical University from November 2018 to October 2020 were examined. The diagnosis of AIS was confirmed by computerized tomography (CT) or magnetic resonance imaging (MRI). The inclusion criteria were age ≥18 years and onset of symptoms ≤ 72 h. The exclusion criteria were patients having active infection on admission, patients with severe hepatic or renal diseases, those who recently underwent major trauma or surgery, cases with a history of malignant tumor, hematologic disease, or immunosuppressive treatments, or those having incomplete medical records. Active infection was defined as preexisting fever or suggestive symptoms including shortness of breath, cough, expectoration, and urinary tract symptoms.

### Data Collection

We recorded all demographic and clinical data, including age, gender, previous history of stroke, hypertension, diabetes, smoking, atrial fibrillation, stroke severity on admission, occurrence of SAI, and laboratory examination values (blood cell counts and albumin levels) within 24 h of hospital admission. Stroke severity was assessed by the National Institutes of Health Stroke Scale (NIHSS). SAI was defined as any new infection occurring within 7 days of stroke onset. Signs of infection such as shortness of breath, cough, expectoration, urinary tract symptoms, and fever were examined by the treating physician as part of the daily ward round. Furthermore, diagnostic workups including laboratory and radiological examinations were also performed at the onset of symptoms. The diagnostic and treatment criteria for SAI were consistent with local clinical practice.

### Statistical Analyses

Statistical analyses were performed using SPSS 19.0 and MedCalc 15.2.2 software. For continuous variables with normal distributions, the data were presented as mean ± standard deviation (SD) and evaluated by independent samples *t*-test. For other distributions, median plus interquartile range (IQR) and the Mann-Whitney *U*-test were used. For categorical variables, the data were presented as frequency and percentage, and evaluated by chi-square test. Furthermore, multivariable logistic regression analysis was performed for the potential confounders (variables with *P* <0.05 in the univariate results). The groups with high and low NPAR were compared by dichotomizing the cohort with median NPAR (1.64) to assess the basic characteristics of subjects with high NPAR. The area under the curve (AUC) of the receiver operating characteristic (ROC) curve was calculated to compare the predictive value of the albumin, neutrophil percentage, NLR, and NPAR. All variables with *P* <0.05 were considered statistically significant.

## Results

This study enrolled 379 patients with AIS out of which 80 (21%) received intravenous thrombolysis. The mean patient age was 68 years and 241 (64%) cases were men. The median NIHSS and NPLR were 2 (1–5) and 1.64 (1.46–1.86), respectively. Out of 379 patients, 51 (13.5%) patients developed SAI, and 29 (57%) experienced fever over 38°C within 7 days after stroke onset. Of all patients with SAI, 34 patients had pneumonia, 12 patients developed urinary tract infections, and 5 patients had other infections.

**Figure 1:**
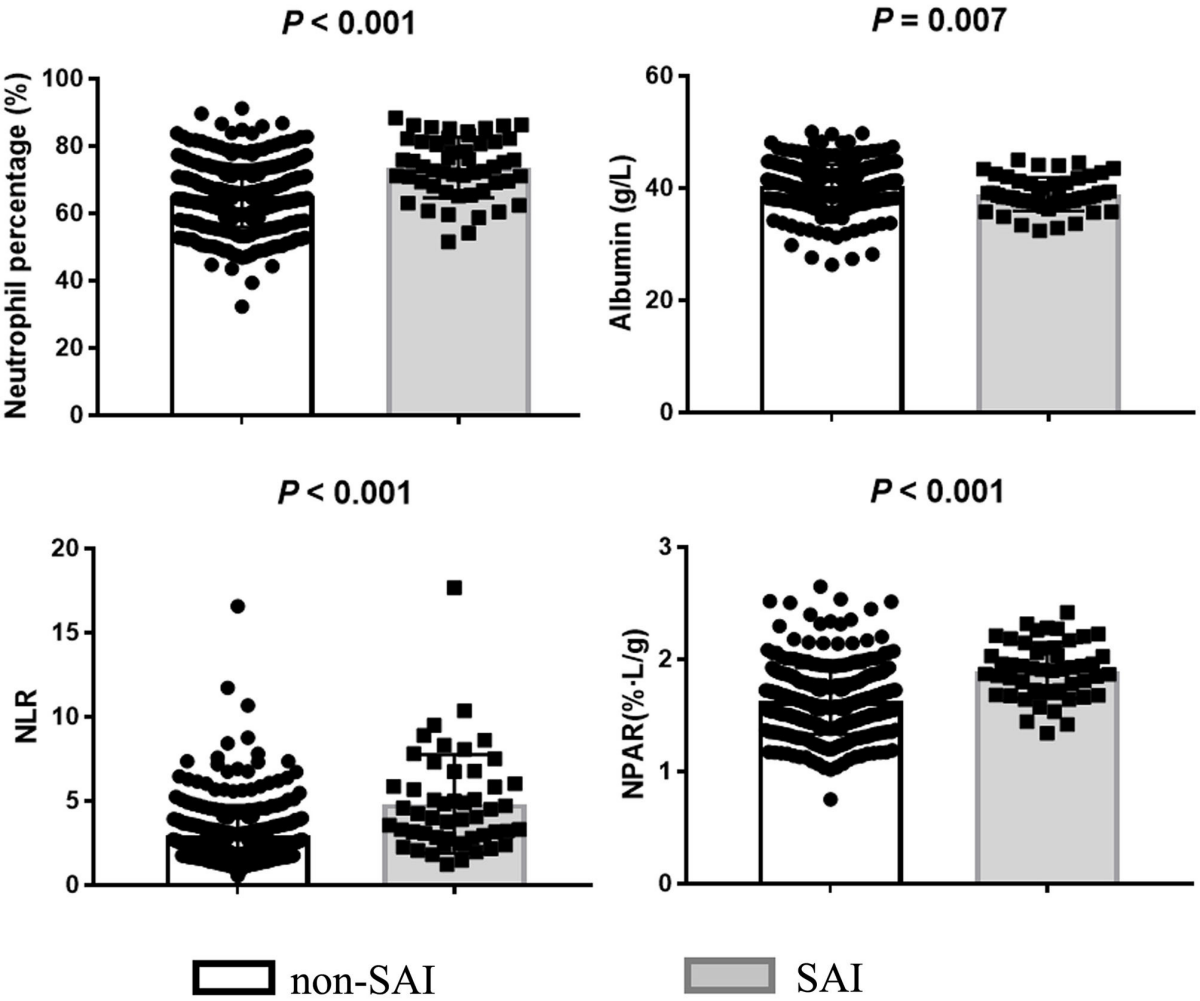
Biomarker levels of neutrophil percentage, albumin, NLR, and NPAR in SAI and non-SAI group.

A comparison between the SAI group (*n* = 51, 13.5%) and the non-SAI group (*n* = 328, 86.5%) revealed no significant difference in the history of stroke, hypertension, and diabetes mellitus. However, patients in the SAI group were found to be older and non-smoking and had a higher proportions of females, atrial fibrillation, and higher initial NIHSS and NLR levels compared to the non-SAI group. In addition, the SAI group presented a significantly higher level of NPAR than that of the non-SAI group [1.91 (1.71–2.09) vs. 1.59 (1.44–1.78); *P* <0.001] (Table 1; Figure 1).

**Table 1:** Baseline characteristics of AIS patients with SAI and non-SAI.

	**Non-SAI (*n* = 328)**	**SAI (*n* = 51)**	***P***
Age (years)	67 ± 12	75 ± 10	<0.001
Male, *n* (%)	222 (68%)	19 (37%)	<0.001
Smoke, *n* (%)	146 (45%)	13 (26%)	0.01
Hypertension, *n* (%)	274 (84%)	41 (80%)	0.577
Diabetes, *n* (%)	129 (39%)	20 (39%)	0.988
Previous stroke, *n* (%)	142 (43%)	21 (41%)	0.776
Atrial fibrillation, *n* (%)	29 (9%)	14 (28%)	<0.001
Initial NIHSS, median (IQR)	2 (1–4)	5 (1–12)	<0.001
Albumin (g/L)	40.4 ± 3.8	38.9 ± 3	0.007
Neutrophil percentage (%)	65.1 ± 9.3	73.6 ± 9.0	<0.001
NLR, median (IQR)	2.50 (1.95–3.48)	4.03 (2.79–6.02)	<0.001
NPAR, median (IQR)	1.59 (1.44–1.78)	1.91 (1.71–2.09)	<0.001

*AIS, acute ischemic stroke; SAI, stroke associated infection; NIHSS, National Institute of Health Stroke Scale; NLR, neutrophil-to-lymphocyte ratio; NPAR, neutrophil percentage-to-albumin ratio; IQR, interquartile range*.

Multivariable logistic regression analysis showed that NPAR remained significant after adjusting for confounders [adjusted odds ratio (aOR) = 10.52; 95% confidence interval (CI), 3.33–33.28; *p* <0.001]. Moreover, male patients (aOR = 0.36; 95% CI, 0.17–0.79; *P* = 0.01) and initial NIHSS (aOR = 1.11; 95% CI, 1.03–1.19; *P* = 0.005) were independently associated with the increased risk of SAI. When sex was excluded from this model, NPAR (aOR = 9.78; 95% CI, 3.14–30.47; *P* <0.001) and NIHSS (aOR = 1.11; 95% CI, 1.04–1.19; *P* = 0.002) remained as independent predictors of SAI (Table 2).

**Table 2:** Multivariable analysis of possible predictors of SAI.

	**Model 1**		**Model 2**	
	**OR (95% CI)**	***P***	**OR (95% CI)**	***P***
Age	1.02 (0.99–1.05)	0.219	1.03 (1.00–1.06)	0.089
Male	0.36 (0.17–0.79)	0.01	—	—
Smoke	1.04 (0.45–2.39)	0.925	0.66 (0.31–1.39)	0.277
Initial NIHSS	1.11 (1.03–1.19)	0.005	1.11 (1.04–1.19)	0.002
Atrial fibrillation	1.43 (0.57–3.57)	0.444	1.34 (0.56–3.25)	0.511
NPAR	10.52 (3.33–33.28)	<0.001	9.78 (3.14–30.47)	<0.001

*SAI, stroke associated infection; NIHSS, National Institute of Health Stroke Scale; NPAR, neutrophil percentage-to-albumin ratio; OR, odds ratio; CI, confidence interval. Model 1: adjusted for age, male, smoke, initial NIHSS, atrial fibrillation, and NPAR. Model 2: adjusted for age, smoke, initial NIHSS, atrial fibrillation, and NPAR*.

A further comparison was conducted between the high and low NPAR groups. As shown in Table 3. The incidence of SAI was higher in the high NPAR group compared to the low NPAR group (23 vs. 3%; *P* <0.001). In addition, patients in the high NPAR group were older and exhibited a higher proportion of female, hypertension, and atrial fibrillation, as well as higher levels of initial NIHSS and NLR than the low NPAR group.

**Table 3:** Baseline characteristics of AIS patients with low and high NPARs.

	**Low NPAR (NPAR ≤ 1.64)**	**High NPAR (NPAR ≥ 1.64)**	***P***
No. of patients	189	189	
SAI, *n* (%)	6 (3%)	44 (23%)	<0.001
Age (years)	65 ± 12	72 ± 11	<0.001
Male, *n* (%)	130 (69%)	111 (59%)	0.042
Smoke, *n* (%)	92 (49%)	66 (35%)	0.007
Hypertension, *n* (%)	150 (79%)	165 (87%)	0.038
Diabetes, *n* (%)	77 (41%)	72 (38%)	0.599
Previous stroke, *n* (%)	79 (42%)	84 (44%)	0.604
Atrial fibrillation, *n* (%)	9 (5%)	34 (18%)	<0.001
Initial NIHSS, median (IQR)	2 (1–4)	3 (1–6)	0.04
Albumin, median (IQR)	41.7 (39.6–44.3)	38.6 (37.2–40.8)	<0.001
Neutrophil percentage (%)	59.8 ± 7.2	72.8 ± 7.1	<0.001
NLR, median (IQR)	2.07(1.59–2.50)	3.62(2.82–5.09)	<0.001

*AIS, acute ischemic stroke; SAI, stroke associated infection; NIHSS, National Institute of Health Stroke Scale; NLR, neutrophil-to-lymphocyte ratio; NPAR, neutrophil percentage-to-albumin ratio; IQR, interquartile range*.

**Figure 2:**
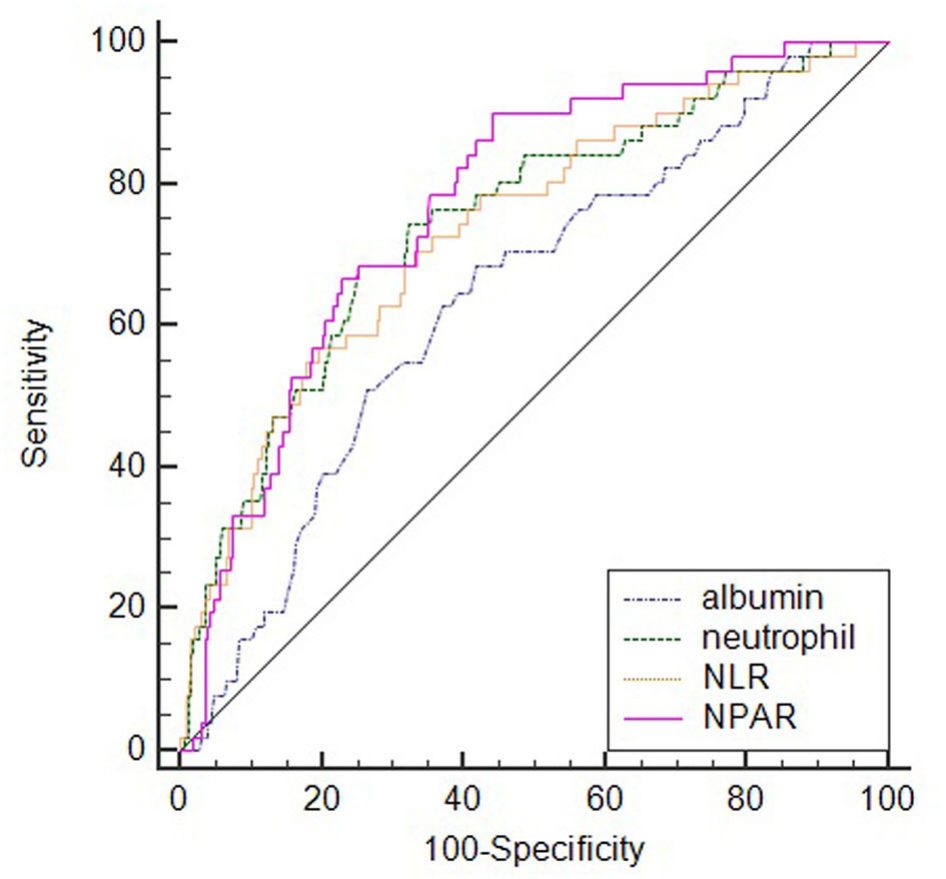
Comparison of predictive value between NPAR and other indicators in the prediction of SAI.

ROC analysis showed the optimal cutoff value of NPAR for predicting SAI was 1.64 with sensitivity and specificity of 90.2 and 55.8%, respectively. While comparing the predictive power with other indicators, NPAR [0.771 (0.725–0.812)] demonstrated the highest AUC value than those of albumin [0.640 (0.590–0.689)], neutrophil percentage [0.747 (0.700–0.790)], and NLR [0.736 (0.689–0.780)]. However, the difference was significant only between NPAR and albumin (*P* = 0.003) (Figure 2).

## Discussion

To our knowledge, this was the first study to explore the diagnostic value of NPAR for SAI; we found that higher NPAR was significantly associated with the risk of SAI occurrence in patients with AIS. More importantly, NPAR may be a more effective biomarker for predicting SAI than albumin, neutrophil percentage, and NLR.

NPAR was a novel marker for systemic inflammation and infection. The elevated NPAR levels may be a result of increased neutrophil percentage and/or decreased albumin levels. In patients with AIS, neutrophils are the first cells to infiltrate the ischemic tissue and their count rises significantly within a few hours (12, 13). To date, a preponderance of data suggests that neutrophils promote blood-brain barrier disruption, cerebral edema, and brain injury (12) which can lead to an accelerated disruption of the homeostatic balance composed of the immune and nervous systems, thereby triggering a local inflammatory immune response and a systemic inflammatory response, including stroke-induced immunosuppression (14–16). Meanwhile, an increase in the peripheral neutrophil is associated with more severe stroke, larger infarct volume, and worse functional outcomes, all of which are strong risk factors for SAI (17–19). Currently, Deng et al. (20) demonstrated that elevated neutrophil was independently associated with increased risk of SAI in AIS patients treated with endovascular therapy. In addition, a ROC curve analysis performed by Nam et al. showed a high AUC value for neutrophils to predict stroke-associated pneumonia (SAP) (21).

As an indispensable substance in various physiological mechanisms, albumin has various functions, such as a major buffer, extracellular antioxidant, immunomodulator, detoxifier, and transporter in plasma (22, 23). Therefore, low serum albumin increases the susceptibility to infection complications. Moreover, hypoalbuminemia, a hallmark of malnutrition, can lead to impaired immune function, pulmonary edema, and fluid retention, thereby promoting the development of infection (24, 25). Additionally, Morotti et al. (26) demonstrated hypoalbuminemia was an independent predictor of pneumonia and sepsis in patients with acute intracerebral hemorrhage. Besides, Dziedzic et al. (27) found serum albumin level to be an independent predictor of SAP in patients with AIS.

Based on the above evidence, we first hypothesized and demonstrated that NPAR, the combination of albumin and neutrophils, showed a good predictive value for the occurrence of SAI. As an indicator that can be implemented even in some underdeveloped medical areas, NPAR is simple, inexpensive, and timely. More importantly, NPAR amplifies the predictive value of neutrophil percentage and albumin, especially when those two do not deviate significantly from the normal range, which often gets overlooked by clinicians. Apparently, NPAR combines the different mechanisms of neutrophil percentage and albumin levels to predict SAI occurrence and displays greater predictive power from the ROC curve. In this study, as shown in Table 3, the high NPAR group also exhibited higher initial NIHSS, as well as higher rates of hypertension and atrial fibrillation than the low NPAR group. As a result, patients with high NPAR might be more likely to develop SAI.

Consistent with previous studies (28, 29), our study showed 13.5% of patients diagnosed with SAI. Further, the AIS patients with high initial NIHSS were more likely to develop SAI, similar to previous studies (1, 21). However, we could not explain the reason for females having a higher risk of developing SAI; besides, we could not rule out that this was a spurious association. When we excluded sex from the statistical model, the results of the analysis did not change significantly, and the aORs for NPAR and NIHSS remained similar to those obtained before. High sensitivity (90.2%) and relatively low specificity (55.8%) were observed when the overall diagnostic value was highest in our study. Therefore, the cutoff value of 1.64 would be more suitable for screening purposes and timely investigation of infection, while a combination of clinical symptoms, and laboratory and radiological examinations would be additionally required to initiate the treatment.

As a widely studied predictor in recent years, elevated NLR has been proved to be highly correlated with the occurrence of SAI or SAP events in patients with AIS (1, 21, 30). In this study, we confirmed this phenomenon, and the predictive value calculated from the ROC curve analysis was also consistent with previous studies (1, 30). While comparing these two indicators NPAR and NLR, we found that NPAR presented a higher predictive value, although the difference was not significant [0.771 (0.725–0.812) vs. 0.736 (0.689–0.780); *P* = 0.1168].

There were several limitations to this study. First, this was a single-center retrospective study and was therefore subjected to selection bias. Further, as there could be racial differences in susceptibility to the occurrence of hypoalbuminemia (31), our findings would need further validation for application in other places. Second, the timing of admission may lead to bias. Although we included AIS patients within 3 days of symptom onset, with nearly 60% of them within 1 day, we may still have missed some SAI events. Furthermore, the activated sympathetic nervous system after stroke mobilizes immune cells from peripheral reservoirs, resulting in an increased number of peripheral immune cells (32), and due to the differences in the time of admission after stroke, this may also poses a challenge to the comparability of NPAR among the patients. Third, due to incomplete data, we did not explore the relationship between NPAR and clinical outcomes in patients with SAI. Gong et al. (22) reported that high NPAR was significantly associated with an increased risk of death in patients with severe sepsis or septic shock. NPAR might indicate a predictive value for the prognosis of SAI. Fourth, although we have tried our best to control the bias with multivariable models, there could be still many other unknown factors influencing our results.

## Data Availability Statement

The original contributions generated for the study are included in the article/supplementary material, further inquiries can be directed to the corresponding author/s.

## Ethics Statement

The studies involving human participants were reviewed and approved by the Ethics Committee of The Second Hospital of Tianjin Medical University. Written informed consent for participation was not required for this study in accordance with the national legislation and the institutional requirements.

## Author Contributions

TW, XT, PL, and JW: collected, analyzed and interpreted the data, and draft the manuscript. HZ and YC: designed and revised the manuscript. All authors contributed to the article and approved the submitted version.

## Conflict of Interest

The authors declare that the research was conducted in the absence of any commercial or financial relationships that could be construed as a potential conflict of interest.

## Publisher's Note

All claims expressed in this article are solely those of the authors and do not necessarily represent those of their affiliated organizations, or those of the publisher, the editors and the reviewers. Any product that may be evaluated in this article, or claim that may be made by its manufacturer, is not guaranteed or endorsed by the publisher.
